# Does Black vs. White race affect practitioners’ appraisal of Parkinson’s disease?

**DOI:** 10.1038/s41531-023-00549-2

**Published:** 2023-07-07

**Authors:** Shana Harris, Nandakumar S. Narayanan, Daniel Tranel

**Affiliations:** 1 Department of Neurology (Division of Neuropsychology and Cognitive Neuroscience), University of Iowa, Iowa City, IA USA; 2 Department of Psychological and Brain Sciences, University of Iowa, Iowa City, IA USA; 3 Departments of Neurology (Division of Neuroscience), University of Iowa, Iowa City, IA USA

**Keywords:** Human behaviour, Parkinson's disease

## Abstract

Black patients are diagnosed with Parkinson’s disease (PD) at half the rate as White patients. The reasons for this large disparity are unknown. Here, we review evidence that practitioner bias may contribute. A key sign of PD is hypomimia or decreased facial expressivity. However, practitioner bias surrounding facial expressivity in Black people versus White people may lead practitioners to appraise Black patients with hypomimia as having higher levels of facial expressivity. Furthermore, practitioner bias may cause them to characterize reduced facial expressivity as being due to negative personality traits, as opposed to a medical sign, in Black patients with hypomimia. This racial bias in the evaluation of hypomimia in Black versus White patients could profoundly impact subsequent referral decisions and rates of diagnosis of PD. Therefore, exploring these differences is expected to facilitate addressing health care disparities through earlier and more accurate detection of PD in Black patients.

Parkinson’s disease (PD) is a neurodegenerative disorder that leads to reduced quality of life, severe disability, and societal cost^1,2^. Parkinson’s disease affects approximately 1 million people in the United States (US), and that number is projected to double in the next 25 years^2^. There is a large racial disparity in the prevalence and incidence of PD in Black and White people^3–5^. “Black” is typically defined as a person having origins in any of the Black racial groups of Africa, whereas “White” is typically defined as a person having origins in any of the original peoples of Europe, the Middle East, or North Africa^6,7^. For example, the prevalence of PD is 50% lower in Black individuals as compared to White individuals, where Black people are half as likely as White people to receive a diagnosis of this disease^3,5^. In the US, the 4-year cumulative incidence of PD has been estimated at 54 per 100,000 among White individuals compared to 23 per 100,000 among Black individuals^4^.

Some factors that might contribute to this large racial disparity in the prevalence and incidence rates of PD in Black and White individuals are genetic or biological differences and systemic and structural factors in health care^3,8^. These factors include access to insurance, patient mistrust in health care, and under-reporting of symptoms. However, in the literature, no significant differences were found between Black and White people on genetic or biological factors^3,9–13^. Of note, these studies often had a low sample size of Black people which affected their generalizability. Hence, larger studies are needed. Regardless, this large disparity in the prevalence and incidence of PD still remains even after controlling for risk factors (demographic and structural) such as age, sex, geography, Medicaid eligibility, and average number of hospital visits^4^. One unexplored possibility is that health-care professionals evaluate motor signs of PD, such as hypomimia (reduced facial expressivity), differently in Black and White patients, a form of practitioner bias. Thus, in this perspective article, we hypothesize that practitioners are under-pathologizing reduced facial expressivity in Black patients with hypomimia. Further, practitioners could be viewing Black people as having higher levels of facial expressivity in comparison to White patients displaying the same level of facial expressivity/severity of hypomimia. In addition, practitioners are likely appraising reduced facial expressivity as being attributed to poor emotional engagement or attitudes, as opposed to a medical pathology, in Black patients with hypomimia in comparison to White patients with the same level of facial expressivity/severity of hypomimia. Indeed, practitioner bias may affect early evaluation of PD patients, leading to under-recognition of early or prodromal signs of PD in Black patients and further perpetuating disparities in diagnosis, treatment, and outcomes for these patients.

## Epidemiology of PD and prodromal motor signs

PD is caused by dysfunction in midbrain dopaminergic neurons, which are important for the production and modulation of movement^14^. A reduction in dopamine neurons contributes to the development of the cardinal motor signs in PD; tremors, rigidity, postural instability, and bradykinesia^15^. Bradykinesia is a key part of many diagnostic criteria and is requisite for diagnosing PD; thus, it is crucial for practitioners to accurately identify this sign in patients^15,16^. Further, hypomimia is one manifestation of bradykinesia^17,18^. Of added importance, hypomimia can be one of the first motor manifestations and is a distinctive clinical feature in patients with PD, presenting as many as 10 years before a clinical diagnosis of PD is determined^17–19^. Hypomimia is present in most patients with PD (approximately 92%; race not reported)^18,20^. Therefore, accurately identifying signs such as hypomimia early in the course of PD could facilitate a timely diagnosis for patients. Further, failure to accurately identify this early sign of PD may result in a delay or false-negative error in the diagnosis of PD and could lead to an underestimation of the prevalence and incidence of this disease in the population, and could have other negative consequences as well.

Epidemiological research has found that the observed prevalence rates of PD are approximately 50% lower in Black patients compared to White patients in the US^3–5^. It is possible that these statistics are an underestimation of the true prevalence and incidence rate of PD among these racial groups. Disease awareness has influenced the diagnosis of other neurological diseases; for instance, the prevalence of multiple sclerosis (MS) in Black people has increased as a result of education of healthcare professionals and the community^21,22^. In fact, the prevalence of other neurological diseases such as MS has increased drastically over the years in Black people – for MS, for example, the crude prevalence rate was 22 per 100,000 in 1998–2000 in comparison to the current age-standardized estimate of 521.4 per 100,000^22^. The low prevalence of MS in previous years could have been the result of underdiagnosis of the disease by practitioners due to bias, among other factors. A similar phenomenon might be occurring with PD.

Prevalence and incidence statistics can be affected by many factors including methodological issues and systemic factors within the health care system^8,23–26^. Schoenberg and colleagues (1985) found that the prevalence rate of PD was not significantly different between Black and White patients within a community in Copiah County, Mississippi (196 vs 280 per 100,000 respectively), after removing healthcare access from the inclusion criteria for their study^26^. Black people tend to have less access to healthcare and are less likely to see neurologists, in comparison to White people^24,27^. Therefore, by removing this factor, researchers were able to include Black patients that were typically underserved in healthcare, and in prevalence and incidence research on PD at that time. They also discovered that twice as many of Black patients with PD had no previous PD diagnoses recorded in their medical records in comparison to Whites, which could indicate that PD signs/symptoms were missed by physicians altogether in many Black patients^26^. Thus, this racial disparity in the evaluation and identification of PD motor signs resulted in a delay or missed diagnosis of PD in Black people, which led to gross underestimations of the true prevalence of this disease within Black patients.

More recent studies have shown that practitioners’ bias related to gender can delay referrals for PD, citing that the duration between the onset of PD signs/symptoms and a referral to a movement disorder specialist was 41% greater in women than in men, after controlling for factors such as time to first primary care visit following PD sign/symptom onset, stage of the disease, age, and family history^28^. Therefore, practitioner’s bias when evaluating motor signs like hypomimia in patients, could contribute to diagnostic delay for PD and, ultimately an underestimation of true prevalence of this disease for Black people.

## Practitioner bias can influence the evaluation of patients with hypomimia

No work has compared the impact of general practitioners’ bias on their evaluations of hypomimia (severity) in Black and White patients with PD. One of the few studies that have investigated the impact of practitioners’ bias on their impressions of mood and traits in patients with PD and hypomimia, used a Caucasian and Asian Taiwanese sample^29^. They found that practitioners were biased in favor of White American patients when evaluating sociability and biased in favor of Asian Taiwanese patients when evaluating cognitive competence and social supportiveness. Practitioners perceived the White patients as being more sociable and Taiwanese patients as being more competent and having more social support. The Tickle-Degnen et. al (2011) study raises the possibility that racial stereotypes are influencing practitioners’ impression and evaluation of Black people with hypomimia^29^.

Practitioners hold stereotypes surrounding facial expressivity in Black people, that can influence their ability to accurately appraise hypomimia in Black patients. White people (both laypersons and experts) have difficulty recognizing the type and magnitude of facial expressions in Black people. Specifically, White people have trouble distinguishing between genuine and false smiles on Black people’s faces^30^. These individuals are also more attuned to the expression and magnitude of anger or negatively valenced emotions on Black faces compared to those on White faces that show a similar intensity of emotion^31–33^. This finding is important because patients with PD and hypomimia appear to exhibit more negatively valenced emotions^29^. Practitioners with this type of bias could potentially view Black patients with PD as having higher levels of emotional expression than White patients with PD, when in fact the levels of expressivity are objectively the same. Consequentially, practitioners might assume that a White patient has more hypomimia impairment than a Black patient, which for the Black patient could lead to a delayed referral to a movement disorder specialist for expert evaluation of possible PD. Ultimately, delays like this could promote large disparities in treatment recommendations and outcomes between Black and White patients.

Delays in treatment of motor and non-motor symptoms in PD can lead to worse outcomes such as falls, decreased quality of life and faster disease progression^34,35^. Researchers have reported that Black patients with PD had higher mortality rates, presented at more advanced stages of PD, and had more motor impairment than White patients with PD after controlling for education and income^36^. These findings further highlight the importance of identifying whether practitioner’s racial bias is leading to a delay in or missed diagnosing of Black patients with hypomimia and PD.

## Practitioner bias can contribute to negative impression of black patients with hypomimia

Bias may also influence practitioners’ impressions of mood and traits in Black patients which can impact their ability to appraise reduced facial expressivity as being a medical sign of PD in these patients. Further, practitioners may misappraise reduced facial expressivity as being a negative personality trait and contribute to negative impressions about attitudes and level of engagement in Black people^37,38^. It is possible that practitioners’ hold stereotypes that influence their impressions of Black people’s attitudes, during medical interviews. The preponderance of these stereotypes has the potential to ultimately impact practitioners’ ability to appraise reduced facial expressivity as a pathological sign of PD in Black people.

Practitioner bias can also affect non-verbal communication with non-white patients^39^. Practitioners view Black patients as being less medically cooperative, risky and more mistrustful than White patients, and reported a greater penchant for working with White patients over Black patients^40,41^. These findings further highlight the possibility that practitioners may form negative impressions about Black patients with hypomimia. This becomes even more significant given that such racial bias can produce differences in interview styles and can affect treatment of Black patients^38,39^. Practitioners may be more likely to characterize reduced facial expressivity as a negative personality trait, such as poor emotional engagement, as opposed to a medical pathology in Black patients than in White patients. Such misappraisal can cause a delay in referral to appropriate care for Black individuals with PD and hypomimia. Evaluating the impact that practitioners’ racial bias has on their appraisal and impressions of facial expressivity might help facilitate timely referrals to specialist care and accurate diagnosis of this disease and better treatment outcomes in Black patients.

## Interventions, limitations, and future directions

Disparities in the prevalence and incidence of PD among Black and White people is multifactorial but may be driven in large part by practitioner bias. Specifically, practitioners’ racial bias could lead to misappraising and under-pathologizing of reduced facial expressivity in Black patients in comparison to White patients, with hypomimia. Exploring sources of health care biases (e.g., practitioner racial bias) in PD may reveal feasible points of intervention for reducing healthcare disparities in PD. For example, targeted intervention techniques such as implicit bias training can reduce biases in practitioners^42,43^. A previous study utilized a multifaceted intervention program, aimed at mitigating the disparities of cancer treatment in Black and White patients, as studies have shown that practitioner’s bias and poor communication leads to treatment variability^42^. However, interventions with training on implicit bias, gatekeeping, and institutional racism for practitioners reduced the disparity in outcomes; indeed, treatment completion in Black patients increased from being 10% lower to being almost equal to White patients^42^. Similar interventions aimed at addressing processes underlying faulty impression formation could attenuate racial disparities in the diagnosis and treatment of medical signs such as hypomimia, as seen in other chronic disorders such as PD^29^.

Another way to reduce health disparities is through fostering trust in healthcare and improving knowledge of PD signs in Black people. Medical mistrust is correlated with patient-perceived discrimination, which is related to their race, income, and insurance. Therefore, multilevel community- and theory-based training models aimed at increasing structural competency (the understanding of the impact of the social structure on a social group or individual) in practitioners can help to mitigate medical mistrust in Black patients and improve engagement in their health care^44,45^.

Increasing health literacy in Black people with PD signs and symptoms will improve self-advocacy, and is another way to help further reduce racial disparities in outcomes for these patients. Health literacy is the ability to obtain and understand health information in order to make informed decisions regarding healthcare and tend to be lower in Black people^46,47^. Further, low health literacy is associated with greater disease severity and morbidity in patients with PD and, in general, is moderated by education and medical mistrust in Black people^46,47^. Therefore, increasing health literacy in these individuals through adopting culturally tailored, interactive, and community-engaged health literacy approaches, would increase Black patients’ ability to advocate for necessary medical resource/services. This is especially important in the context of PD since Black people are less likely to see neurologists, in comparison to White people^24,27^. Overall, improving health literacy in Black patients will aid in their ability to understand and advocate for their health, especially when practitioner’s bias is impacting the medical encounter.

### Limitation

One limitation to this viewpoint is that there may be genetic or biological factors that are driving differences in PD prevalence between Black and White people. As noted, there are few studies on this topic with an adequate sample size. Therefore, PD research equity may result from increased patient awareness of clinical research opportunities, increased availability of culturally diverse research materials, and partnership with community organizations^48^. Additionally, targeted training may reduce racial disparities in practitioners’ referrals of Black patients to clinical trials^48^. Identifying the sources of racial disparities in PD will be highly clinically meaningful.

### Future direction

Future research will explore ways in which practitioner bias impacts their evaluation of advanced motor and non-motor signs of PD in patients from other racially diverse groups, such as Asian people, given the lower prevalence of PD in this population in comparison to their White counterparts^5^. In addition, researchers can investigate whether factors such as practitioners’ race or years of experience in healthcare moderate their ability to accurately appraise PD signs, such as hypomimia, in Black patients. This line of work is significant as it will aid in the development of intervention techniques and policies aimed at reducing health disparities for Black individuals with PD and other movement disorders.

### Reporting summary

Further information on research design is available in the Nature Research Reporting Summary linked to this article.

## Supplementary information

Reporting Summary

## Data Availability

Data sharing is not applicable to this article as no datasets were generated or analyzed for the current article.

## References

[1] Chen JJ (2010). Parkinson’s disease: Health-related quality of life, economic cost, and implications of early treatment. Am. J. Manag Care..

[2] Yang W (2020). Current and projected future economic burden of Parkinson’s disease in the U.S. NPJ Park Dis..

[3] Bailey M, Anderson S, Hall DA (2020). Parkinson’s disease in African Americans: a review of the current literature. J. Parkinsons Dis..

[4] Dahodwala N (2009). Racial differences in the diagnosis of Parkinson’s disease. Mov. Disord..

[5] Wright Willis A, Evanoff BA, Lian M, Criswell SR, Racette BA (2010). Geographic and ethnic variation in Parkinson disease: a population-based study of US Medicare beneficiaries. Neuroepidemiology.

[6] Bhopal R, Donaldson L (1998). White, European, Western, Caucasian, or what? Inappropriate labeling in research on race, ethnicity, and health. Am. J. Public Health.

[7] Flanagin A, Frey T, Christiansen SL, AMA Manual of Style Committee. (2021). Updated guidance on the reporting of race and ethnicity in medical and science journals. JAMA.

[8] Dahodwala N, Xie M, Noll E, Siderowf A, Mandell DS (2009). Treatment disparities in Parkinson’s disease. Ann. Neurol..

[9] Jain S (2011). The risk of Parkinson disease associated with urate in a community-based cohort of older adults. Neuroepidemiology.

[10] Maynard JW (2014). Racial differences in gout incidence in a population-based cohort: atherosclerosis risk in communities study. Am. J. Epidemiol..

[11] McGuire V (2011). Association of DRD2 and DRD3 polymorphisms with Parkinson’s disease in a multiethnic consortium. J. Neurol. Sci..

[12] McInerney-Leo A, Gwinn-Hardy K, Nussbaum RL (2004). Prevalence of Parkinson’s disease in populations of African ancestry: a review. J. Natl Med. Assoc..

[13] Olsen JH, Friis S, Frederiksen K (2006). Malignant melanoma and other types of cancer preceding Parkinson disease. Epidemiology.

[14] Wichmann T, Dostrovsky JO (2011). Pathological basal ganglia activity in movement disorders. Neuroscience.

[15] Postuma RB (2015). MDS clinical diagnostic criteria for Parkinson’s disease. Mov. Disord..

[16] Tarakad A, Jankovic J (2017). Diagnosis and management of Parkinson’s disease. Semin. Neurol..

[17] Maycas-Cepeda T (2021). Hypomimia in Parkinson’s disease: what is it telling us. Front. Neurol..

[18] Novotny M (2022). Automated video-based assessment of facial bradykinesia in de-novo Parkinson’s disease. NPJ Digit. Med..

[19] Fereshtehnejad SM, Skogar Ö, Lökk J (2017). Evolution of orofacial symptoms and disease progression in idiopathic Parkinson’s disease: longitudinal data from the Jönköping Parkinson Registry. Parkinson’s Dis..

[20] Ricciardi L (2020). Hypomimia in Parkinson’s disease: an axial sign responsive to levodopa. Eur. J. Neurol..

[21] Amezcua L, Rivera VM, Vazquez TC, Baezconde-Garbanati L, Langer-Gould A (2021). Health disparities, inequities, and social determinants of health in multiple sclerosis and related disorders in the US: a review. JAMA Neurol..

[22] Langer-Gould AM, Gonzales EG, Smith JB, Li BH, Nelson LM (2022). Racial and ethnic disparities in multiple sclerosis prevalence. Neurology.

[23] Dahodwala N, Willis AW, Li P, Doshi JA (2016). Prevalence and correlates of anti-Parkinson drug use in a nationally representative sample. Mov. Disord. Clin. Pract..

[24] Saadi A, Himmelstein DU, Woolhandler S, Mejia NI (2017). Racial disparities in neurologic health care access and utilization in the United States. Neurology.

[25] Schneider MG (2009). Minority enrollment in Parkinson’s disease clinical trials. Parkinsonism Relat. Disord..

[26] Schoenberg BS, Anderson DW, Haerer AF (1985). Prevalence of Parkinson’s disease in the biracial population of Copiah County, Mississippi. Neurology.

[27] Shi L, Chen CC, Nie X, Zhu J, Hu R (2014). Racial and socioeconomic disparities in access to primary care among people with chronic conditions. J. Am. Board Fam. Med..

[28] Saunders-Pullman R, Wang C, Stanley K, Bressman SB (2011). Diagnosis and referral delay in women with Parkinson’s disease. Gend. Med..

[29] Tickle-Degnen L, Zebrowitz LA, Ma HI (2011). Culture, gender and health care stigma: practitioners’ response to facial masking experienced by people with Parkinson’s disease. Soc. Sci. Med..

[30] Friesen JP (2019). Perceiving happiness in an intergroup context: the role of race and attention to the eyes in differentiating between true and false smiles. J. Pers. Soc. Psychol..

[31] Hugenberg K, Bodenhausen GV (2003). Facing prejudice: Implicit prejudice and the perception of facial threat. Psychol. Sci..

[32] Hugenberg K, Bodenhausen GV (2004). Ambiguity in social categorization: the role of prejudice and facial affect in race categorization. Psychol. Sci..

[33] Hutchings PB, Haddock G (2008). Look Black in anger: the role of implicit prejudice in the categorization and perceived emotional intensity of racially ambiguous faces. J. Exp. Soc. Psychol..

[34] Merola A (2015). Earlier versus later subthalamic deep brain stimulation in Parkinson’s disease. Parkinsonism Relat. Disord..

[35] Parkinson Study Group. (2004). A controlled, randomized, delayed-start study of Rasagiline in early Parkinson disease. Arch. Neurol..

[36] Dahodwala N, Karlawish J, Siderowf A, Duda JE, Mandell DS (2011). Delayed Parkinson’s disease diagnosis among African-Americans: the role of reporting of disability. Neuroepidemiology.

[37] Hagiwara N, Dovidio JF, Eggly S, Penner LA (2016). The effects of racial attitudes on affect and engagement in racially discordant medical interactions between non-Black physicians and Black patients. Group Process Intergroup Relat..

[38] Hall WJ (2015). Implicit racial/ethnic bias among health care professionals and its influence on health care outcomes: A systematic review. Am. J. Public Health.

[39] Levine CS, Ambady N (2013). The role of non-verbal behaviour in racial disparities in health care: implications and solutions. Med Edu..

[40] Oliver MN, Wells KM, Joy-Gaba JA, Hawkins CB, Nosek BA (2014). Do physicians’ implicit views of African Americans affect clinical decision making?. J. Am. Board Fam. Med..

[41] van Ryn M, Burke J (2000). The effect of patient race and socio-economic status on physicians’ perceptions of patients. Soc. Sci. Med..

[42] Cykert S (2020). A multi-faceted intervention aimed at Black-White disparities in the treatment of early stage cancers: the ACCURE pragmatic quality improvement trial. J. Natl Med Assoc..

[43] Kawakami K, Dovidio JF, Moll J, Hermsen S, Russin A (2000). Just say no (to stereotyping): effects of training in the negation of stereotypic associations on stereotype activation. J. Pers. Soc. Psychol..

[44] Bazargan M, Cobb S, Assari S (2021). Discrimination and medical mistrust in a racially and ethnically diverse sample of California adults. Ann. Fam. Med..

[45] Wang EE (2019). Structural Competency: What is it, why do we need it, and what does the structurally competent emergency physician look like?. AEM Educ. Train..

[46] Fleisher JE, Shah K, Fitts W, Dahodwala NA (2016). Associations and implications of low health literacy in Parkinson’s Disease. Mov. Disord. Clin. Pract..

[47] Muvuka B (2020). Health literacy in African-American communities: barriers and strategies. Health Lit. Res Pract..

[48] Adrissi J, Fleisher J (2022). Moving the dial toward equity in Parkinson’s disease clinical research: a review of current literature and future directions in diversifying PD clinical trial participation. Curr. Neurol. Neurosci. Rep..

